# Linking behavioral thermoregulation, boldness, and individual state in male Carpetan rock lizards

**DOI:** 10.1002/ece3.6685

**Published:** 2020-08-17

**Authors:** Gergely Horváth, Octavio Jiménez‐Robles, José Martín, Pilar López, Ignacio De la Riva, Gábor Herczeg

**Affiliations:** ^1^ Behavioural Ecology Group Department of Systematic Zoology and Ecology Eötvös Loránd University Budapest Hungary; ^2^ Department of Ecology and Evolution Research School of Biology Australian National University Canberra Australia; ^3^ Department of Biodiversity and Evolutionary Biology Museo Nacional de Ciencias Naturales CSIC Madrid Spain; ^4^ Department of Evolutionary Ecology Museo Nacional de Ciencias Naturales CSIC Madrid Spain

**Keywords:** animal personality, *Iberolacertacyreni*, individual state, thermal biology, thermoregulatory strategy

## Abstract

Mechanisms affecting consistent interindividual behavioral variation (i.e., animal personality) are of wide scientific interest. In poikilotherms, ambient temperature is one of the most important environmental factors with a direct link to a variety of fitness‐related traits. Recent empirical evidence suggests that individual differences in boldness are linked to behavioral thermoregulation strategy in heliothermic species, as individuals are regularly exposed to predators during basking. Here, we tested for links between behavioral thermoregulation strategy, boldness, and individual state in adult males of the high‐mountain Carpetan rock lizard (*Iberolacerta cyreni*). Principal component analysis revealed the following latent links in our data: (i) a positive relationship of activity with relative limb length and color brightness (PC1, 23% variation explained), (ii) a negative relationship of thermoregulatory precision with parasite load and risk‐taking (PC2, 20.98% variation explained), and (iii) a negative relationship between preferred body temperature and relative limb length (PC3, 19.23% variation explained). We conclude that differences in boldness and behavioral thermoregulatory strategy could be explained by both stable and labile state variables. The moderate link between behavioral thermoregulatory strategy and risk‐taking personality in our system is plausibly the result of differences in reproductive state of individuals or variation in ecological conditions during the breeding season.

## Graphical abstract

Body temperature is a major ecophysiological variable in poikilotherms with a direct link to a variety of fitness‐related traits. As most poikilotherms are capable of maintaining relatively high and constant body temperatures by behavioural adjustments, we tested whether behavioural thermoregulatory strategy are linked to boldness and individual state in adult males of the high mountain Carpetan rock lizard (*Iberolacerta cyreni*). Our results suggest that even though there is a moderate link between behavioural thermoregulation strategy and among‐individual variation in risk‐taking in this species, behavioural variation is primarily affected by individual state.

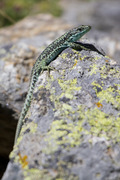

## 1 INTRODUCTION

Heritable interindividual phenotypic variation within a population is essential for adaptive evolutionary change (Botero, Weissing, Wright, & Rubenstein, 2015; Wagner & Wagner, 1996; West‐Eberhard, 1989). Behavior is perhaps the most plastic phenotypic trait (see West‐Eberhard, 2003), which is the main reason why biological and evolutionary significance of consistent interindividual behavioral variation within populations (i.e., animal personality) has been underappreciated for a long time (see Dall, Houston, & McNamara, 2004; Gosling, 2001; Réale, Reader, Sol, McDougall, & Dingemanse, 2007; Sih, Bell, & Johnson, 2004; Sih, Bell, Johnson, & Ziemba, 2004). Nevertheless, studies targeting animal personality have become more common (for reviews and meta‐analyses see Brommer & Class, 2017; Garamszegi, Markó, & Herczeg, 2013; Jandt et al., 2014; Sih et al., 2015; Smith & Blumstein, 2008; Webster & Ward, 2011). Related research revolves around the question of how consistent between‐individual differences emerge against behavioral flexibility (see West‐Eberhard, 2003). A large number of conceptual and empirical studies suggest that consistent between‐individual behavioral differences are linked to differences in both inherently stable (e.g., size, sex differences, brain structure) and labile (e.g., energy reserves, health state, reproductive value) features of individual state (see DiRienzo, Niemelä, Hedrick, & Kortet, 2016; Horváth, Martín, López, Garamszegi, & Herczeg, 2017; Lichtenstein et al., 2016; Luttbeg & Sih, 2010; Mathot & Dall, 2013; Mathot, Dekinga, & Piersma, 2017; Urszán, Török, Hettyey, Garamszegi, & Herczeg, 2015).

In poikilotherms (i.e., animals whose internal temperature varies considerably), body temperature influences virtually all biological processes associated with fitness and behavior (Abram, Boivin, Moiroux, & Brodeur, 2017; Adolph & Porter, 1993; Angilletta, 2001; Huey & Bennett, 1987; Nylin & Gotthard, 1998). Most poikilotherms are capable of maintaining relatively high and constant body temperatures (Cowles & Bogert, 1944) by physiological and (mainly) behavioral adjustments (Angilletta, Cooper, Schuler, & Boyles, 2002; Herczeg, Gonda, Saarikivi, & Merilä, 2006; Herczeg et al., 2008; Huey & Slatkin, 1976; Stevenson, 1985; Van Damme & Bauwens, 1991). To attain and maintain given body temperature, it is important to maximize the efficiency of various physiological and behavioral processes. Thus, one would expect a strong directional selection toward lower interindividual variation in thermal preferences (the “goal” of thermoregulation), but empirical evidence suggests that selected body temperatures might consistently differ between individuals (Angilletta, Hill, & Robson, 2002; Goulet, Thompson, & Chapple, 2017; Stapley, 2006). Further, a growing number of empirical studies suggest a link between components of thermoregulatory strategy and behavioral consistency in poikilotherms. Despite some results indicating a lack of a general pattern of relationship between behavioral thermoregulatory strategy and personality traits (Artacho, Jouanneau, & Le Galliard, 2013; Cerqueira et al., 2016; Goulet, Ingley, Scharf, & Pruitt, 2016; Goulet, Thompson, & Chapple, 2017; Goulet, Thompson, Michelangeli, Wong, & Chapple, 2017; Herrel, James, & Van Damme, 2007; Michelangeli, Goulet, Kang, Wong, & Chapple, 2018; Rey, Digka, & MacKenzie, 2015; Stapley, 2006), lower behavioral predictability (i.e., intraindividual behavioral variation, see Stamps, Briffa, & Biro, 2012) seems to be associated with high ambient temperatures (≈ body temperature) in various poikilotherm taxa (Briffa, Bridger, Biro, & a., 2013; Nakayama, Laskowski, Klefoth, & Arlinghaus, 2016; Velasque & Briffa, 2016).

Ecology of thermoregulation is well studied in reptiles (see Bajer, Molnár, Török, & Herczeg, 2012; Bauwens, Hertz, & Castilla, 1996; Berkel & Clusella‐Trullas, 2018; Herczeg et al., 2004, 2006, 2008; Mészáros, Herczeg, Bajer, Török, & Molnár, 2018; Rusch & Angilletta, 2016), especially in small‐ to medium‐sized heliothermic lizards, whose body temperature is maintained by behavioral (timing of activity, microhabitat use, adopted posture) rather than by physiological adjustments (Angilletta, Cooper, et al., 2002; Bauwens et al., 1996; Huey & Slatkin, 1976; Van Damme & Bauwens, 1991). Differences in individual thermal preferences seem to play a key role for behavioral consistency in reptiles (Goulet, Thompson, Michelangeli, et al., 2017; Mell et al., 2016; Stapley, 2006; Waters, Bowers, & Burghardt, 2017). In addition, differences in time available for thermoregulation (irrespectively of body temperature) could affect behavioral consistency at different levels (Horváth, Mészáros, et al., 2017). As active behavioral thermoregulation has several costs in lizards (e.g., increased risk of predation; Huey & Slatkin, 1976), thermal preferences are particularly expected to be affected by boldness. Recently, Goulet, Thompson, and Chapple (2017) and Michelangeli et al. (2018) reported the existence of individual “thermal types” in Delicate skinks (*Lampropholis delicata*), with individuals preferring higher body temperatures also showing higher scores of locomotor performance, activity, exploration, and risk‐taking. Similar patterns were found in Namib rock agamas (*Agama planiceps*) (Carter, Goldizen, & Tromp, 2010), and Eastern box turtles (*Terrapene carolina*) (Kashon & Carlson, 2018). Since behavioral thermoregulatory strategy is expected to depend on the individual state (e.g., size, health), more studies are needed to seek links between thermoregulatory strategy, other personality traits, and physiological state of individuals.

Here, we aimed to test for associations between behavioral thermoregulatory strategy, boldness, and individual state in Carpetan rock lizards (*Iberolacerta cyreni*), a medium‐sized lacertid endemic to mountain ranges in the center of the Iberian Peninsula. Species in the genus *Iberolacerta* are cold‐adapted lizards living mainly in low thermal quality environments (Aguado & Braña, 2014; Carrascal, López, Martín, & Salvador, 1992; Jiménez‐Robles & De la Riva, 2019; Monasterio, Salvador, Iraeta, & Díaz, 2009; Ortega, Federal, & Grosso, 2017; Ortega, Mencía, & Pérez‐Mellado, 2017; Žagar, Carretero, Osojnik, Sillero, & Vrezec, 2015). Recent empirical data on *I. cyreni* indicate the existence of animal personality in different behavioral traits, with short‐term differences in state and environment potentially contributing to between‐individual behavioral variation (Horváth et al., 2016; Horváth, Martín, et al., 2017; Horváth, Mészáros, et al., 2017; López, Hawlena, Polo, Amo, & Martín, 2005). However, whether consistent individual differences are present in thermoregulatory strategy and whether these are linked to other personality traits in *I. cyreni* are virtually unknown. Here, we studied the potential connections between activity and risk‐taking personality, preferred body temperature, thermoregulatory precision, and various fitness‐linked state variables (body size, relative limb length, color, blood parasite load) applying seminatural and laboratory experiments. Previous empirical results indicate that individuals with proactive personalities (i.e., more active and risk‐prone) select higher body temperatures (Cerqueira et al., 2016; Michelangeli et al., 2018; Stapley, 2006); thus, we expected that bolder individuals would have higher preferred body temperature and higher thermoregulatory precision (i.e., keeping body temperature within a narrow range). It is plausible that different state variables might either constrain or promote thermoregulatory precision, but because the direction of such relationships is difficult to predict, and relevant empirical studies are scarce, we do not have explicit predictions.

## 2 MATERIALS AND METHODS

### 2.1 Collection and housing

**Figure 1:**
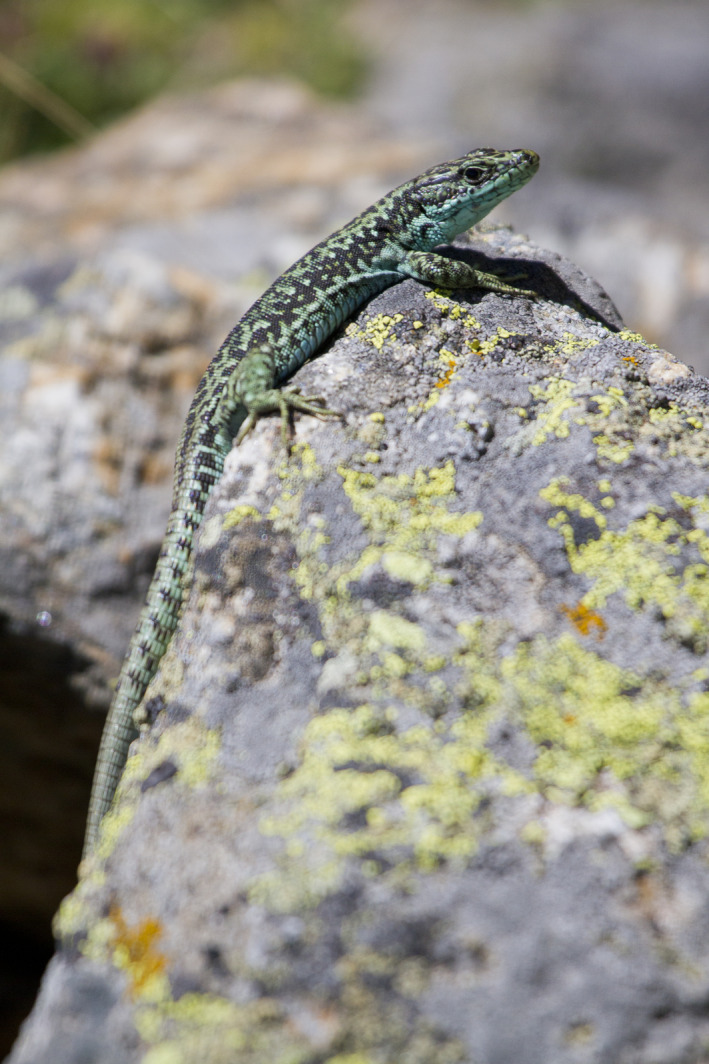
Adult male Carpetane rock lizard (*Iberolacerta cyreni*). Photograph by Octavio Jiménez‐Robles

The study used 24 adult male lizards (Figure 1) whose behavior had previously been scored (Horváth et al., 2016). They were captured during early June 2013 at the slopes of “Alto del Telégrafo” (Sierra de Guadarrama National Park, Madrid Province, Spain, 1,900 m asl, approximately). Individuals were transported to the “El Ventorrillo” Field Station (1,500 m asl, approximately), 5 km downhill from the capture site, where they were housed individually outdoors in opaque plastic boxes (56.5 cm × 36.5 cm × 31.4 cm; length, width, height, respectively). In the boxes, we used a thin layer of coconut fiber as substrate and provided a hollow brick as shelter. Water and food (House crickets [*Acheta domesticus*] and Turkestan cockroaches [*Blatta lateralis*]) were provided ad libitum during captivity. All lizards were released at the original capture site after the experiments.

### 2.2 Individual traits

Upon capture, we measured morphological and color traits, and took blood from each individual to quantify the intensity of blood parasite infection. Snout–vent length (hereafter SVL; 65.52 ± 3.2; mean ± *SD*) and length of the hind limbs were measured using a digital calliper to the nearest 0.01 mm. Hind limb length was characterized by measuring the left and right femurs and tibias, and then summing the mean femur (10.35 ± 0.5; mean ± *SD*) and tibia lengths (10.66 ± 0.4; mean ± *SD*) for every individual. In the analyses (see below), we used residuals from the hind limb length–SVL regression to describe relative hind limb length (hereafter limb length).

We used 25‐G insulin syringes to take blood from a large subcutaneous vessel on the ventral side of each individual. Blood was collected using 60 µl hematocrit capillary tubes, and blood smears were made by blowing a drop of blood onto a microscope slide. Smears were air‐dried until coagulation, then fixed with methanol and stained. For a detailed description of the process, see Molnár, Bajer, Mészáros, Török, and Herczeg (2013). Data on *Karyolysus* and *Schellackia* infection intensity (hereafter parasite load; 18 ± 25.87; mean ± *SD*) were quantified under the microscope at x1000 magnification and normalized using log_10_ transformation. Only one individual was found uninfected (retained in the analyses); hence, parasite prevalence was 0.95.

Reflectance was measured on the animals’ green‐colored dorsal scales using a spectrometer (USB2000 Ocean Optics, Dunedin, FL, USA) with a deuterium‐halogen light source (DT‐MINI‐2GS, OceanOptics). Three different measures were taken from random spots between the 300–700 nm wavelength range. We took the average of every 5 nm; then, we calculated the average of the three measurements. A principal component analysis (PCA) was run on the spectrum data to gather new variables describing dorsal coloration (see Cuthill, Bennett, Partridge, & Maier, 1999; Grill & Rush, 2000; Kopena, López, & Martín, 2014). Based on Kaiser–Guttmann criterion, principal components (PCs) with eigenvalue greater than 1 were retained (Tabachnick & Fidell, 2014). As a result, we obtained a single PC describing the total achromatic brightness of the dorsal scales (hereafter dorsal brightness; proportion of variation explained = 88.41%; factor loadings > 0.982; see Table S1).

### 2.3 Behavioral assays

Methodology of behavioral assays is described in detail in Horváth et al. (2016). Briefly, activity and risk‐taking of each individual were tested 5 and 6 times (respectively) over a 13‐day period between 13 and 25 June 2013 on alternating days (an activity assay was skipped on 19 June because of thick cloud cover and light rain). Activity was measured in the animals’ home cages by analyzing video recordings and was represented by total distance moved during three 10‐min time slots in each trial. Risk‐taking was measured on every second day in unfamiliar arenas (36.5 cm × 22.4 cm × 25 cm; length, width, height, respectively). First, animals were handled by the experimenter and placed into a smaller “starter box” (11.2 cm × 7.3 cm × 5.4 cm; length, width, height, respectively). Then, after a 5‐min acclimation period, the door of the starter box was opened and the latency time to emerge was recorded for 10 min. Out of 120 assays, individuals did not leave the refuge in 16 cases (13.3% of all assays). These observations received maximum score. Both activity and risk‐taking behaviors were found repeatable (activity: r = 0.69, 95% confidence interval [CI] =0.51 – 0.8; risk‐taking: r = 0.22, 95% CI = 0.11–0.41), indicating the presence of animal personality (Horváth et al., 2016). However, there was no significant between‐individual correlation between the behaviors (Horváth et al., 2016), indicating the lack of a “true” behavioral syndrome (see Dingemanse, Dochtermann, & Wright, 2010; Herczeg & Garamszegi, 2012). Hence, both activity and risk‐taking can be seen as independent traits with clear between‐individual differences. Here, we used the individual means to describe individual behavioral type (hereafter activity and risk‐taking).

### 2.4 Thermoregulatory strategy

After the behavioral tests, on 27 and 29 June, we measured thermal preferences of the study individuals (using the same setup as in Jiménez‐Robles & De la Riva, 2019). We intended to measure voluntary thermal preference in an ecological cost‐free environment (Hertz, Huey, & Stevenson, 1993), where the only varying environmental factor is the temperature gradient. Hence, experiments were run in a simplified, special enclosure under controlled conditions to minimize confounding factors (e.g., differences in solar radiation and wind, uncontrolled background temperature, heterogeneity of substrates and their thermal properties, etc). We built 12 tracks (100 cm × 16.5 cm × 45 cm; length, width, height, respectively) using smoothened particle board, following a similar laboratory setup as in Paranjpe et al. (2014). With an incandescent light bulb (100 W) over one end of the track and aluminum foil covering the surfaces of walls and top of each bulb, we created a temperature gradient from more than 50°C to <25°C. Thermal ecophysiology in poikilotherms is typically framed around the concept of core body temperatures (intracloacal in lizards), a thermocouple (0.076 mm diameter, 40 AWG gauge; Omega 5SC‐TT‐T‐40‐36) with nail polish‐coated tip (1 mm approximately of final diameter), was inserted 1 cm deep into the cloaca and taped around the tail base of lizards (Aguado & Braña, 2014; Gvoždík, Castilla, & Gvozdik, 2001; Herrando‐Pérez et al., 2019). We placed one lizard in one track. Each thermocouple was connected to a multichannel data logger (Eltek Squirrel 1035, Eltek Ltd., Cambridge, UK) to record body temperatures once per minute for ~2 hr. We discarded the first measures until the lizards could reach the first maximum in their sine‐like thermoregulatory pattern. We also discarded measures when the thermocouples got outside the cloaca or when the lizards entangled in the wire without possibility to move freely (Aguado and Braña, 2016; Jiménez‐Robles & De la Riva, 2019). In spite of potential stress effects, intracloacal thermocouples provide the most accurate method for thermoregulatory measures for lizards (Garcia‐Porta et al., 2019; Sinervo et al., 2010). Nevertheless, as the setup was the same for all the lizards, observed differences in the experiment should reflect differences in personality of thermoregulation.

### 2.5 Statistical analyses

We calculated the median of selected body temperatures (*T*_sel_); the voluntary thermal maximum (*T*_Vmax_), defined as the highest body temperature, reached during the experiment, as a measure of how much every individual dares to approach potentially deleterious high temperatures, which are usually slightly above the optimum temperature; and the width of the setpoint range (*T*_set_) defined as the central 50% of recorded body temperatures as a measure of the precision of thermoregulatory strategy. We note that applying mean and standard deviation of selected body temperatures as alternative indices of thermoregulatory strategy did not change our results qualitatively. Hence, apart from the *T*_Vmax_, we chose to stick with T_sel_ and T_set_, as these are more frequently used measures of thermal biology in reptiles. We note that the thermal measurements failed in three individuals and data inspection revealed two additional individuals being extreme outliers (one in selected temperature and one in body size); hence, we excluded these individuals from the analyses.

We should note here that due to laboratory setup capacity, individuals were measured at different time of day during two different days. In order to test whether these differences affected selected temperatures, we ran a general linear mixed model (LMM) with recorded body temperatures as response variable, different dates and time of day and their interaction as fixed effects, and individual identity as a random factor. We applied backward stepwise model selection of all effects of the LMM using the *step* function available via the *lme4* and *lmerTest* packages (Bates, Mächler, Bolker, & Walker, 2015; Kuznetsova, Brockhoff, & Christensen, 2016). The significance of the fixed effects was estimated based on Satterthwaite approximation, while likelihood‐ratio test was used for the random effects.

Our main goal was to look for relationships among personality traits (activity, risk‐taking), traits describing individual thermoregulatory strategy (*T*_sel_, *T*_Vmax_, *T*_set_) and traits describing individual state (SVL, relative limb length, parasite load, dorsal brightness). In this framework, there are no predictor or response variables. Hence, we applied a PCA to find the “simple structure” behind the variables or, in other words, to find the latent relationships in the data. In this approach, the best original variable—PC correlations—is the achievable goal, which can be reached by rotation techniques. Further, unlike in cases where PCA is used for reducing the number of original variables by collapsing them into independent PCs, here the independence of the PCs is not necessary, because the best simple structure might be found with correlated PCs. Therefore, we used the following strategy. First, we ran our PCA with an oblique rotation (promax) allowing correlations among PCs. Since the correlation coefficients were relatively low (*r* < |0.2|), we reran the PCA with an orthogonal rotation (varimax) resulting in independent PCs. We note that the loading structure of the two PCAs was qualitatively similar and we report the solution after varimax rotation (Robinson et al., 2018; Schuett & Dall, 2010; Tab achnick & Fidell, 2014). Bartlett tests were significant, indicating that the correlation matrices were significantly different from the identity matrices. Anti‐image correlations were mostly low (see Table S2). PCs with an eigenvalue greater than 1 were retained, based on Kaiser–Guttmann criterion. This initial approach resulted in four PCs, the fourth PC having less than three variables loading on it. Since PCs with less than three original variables loading on them are unreliable and should be discarded (Tabachnick & Fidell, 2014), we reran the analysis and extracted the first three PCs only. For interpreting factor loadings, following the suggestions of Comrey and Lee (2009), Tabachnick and Fidell (2014) recommended a loading above 0.71 (50% overlapping variance) to be considered excellent, 0.63 (40% overlapping variance) very good, 0.55 (30% overlapping variance) good, 0.45 (20% overlapping variance) fair, 0.32 (10% overlapping variance) poor, and anything below 0.32 uninterpretable. However, due to our relatively low sample size, we only interpreted loadings above 0.45. All data analysis was conducted in R version 3.6.2 (R Developmental Core Team, 2019).

## 3 RESULTS

**Figure 2:**
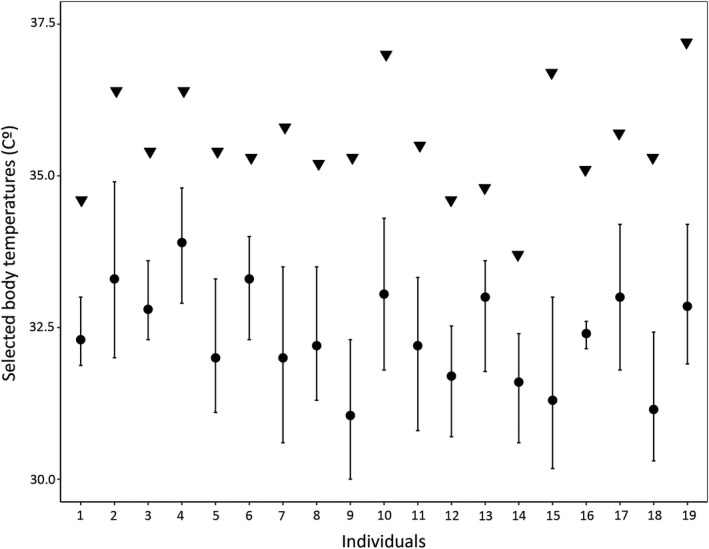
Individual differences in selected body temperatures of adult male *Iberolacerta cyreni*. Median of selected body temperatures (represented as dots) with setpoint range (central 50%) and voluntary maximum temperatures (represented as triangles) are shown

**Figure 3:**
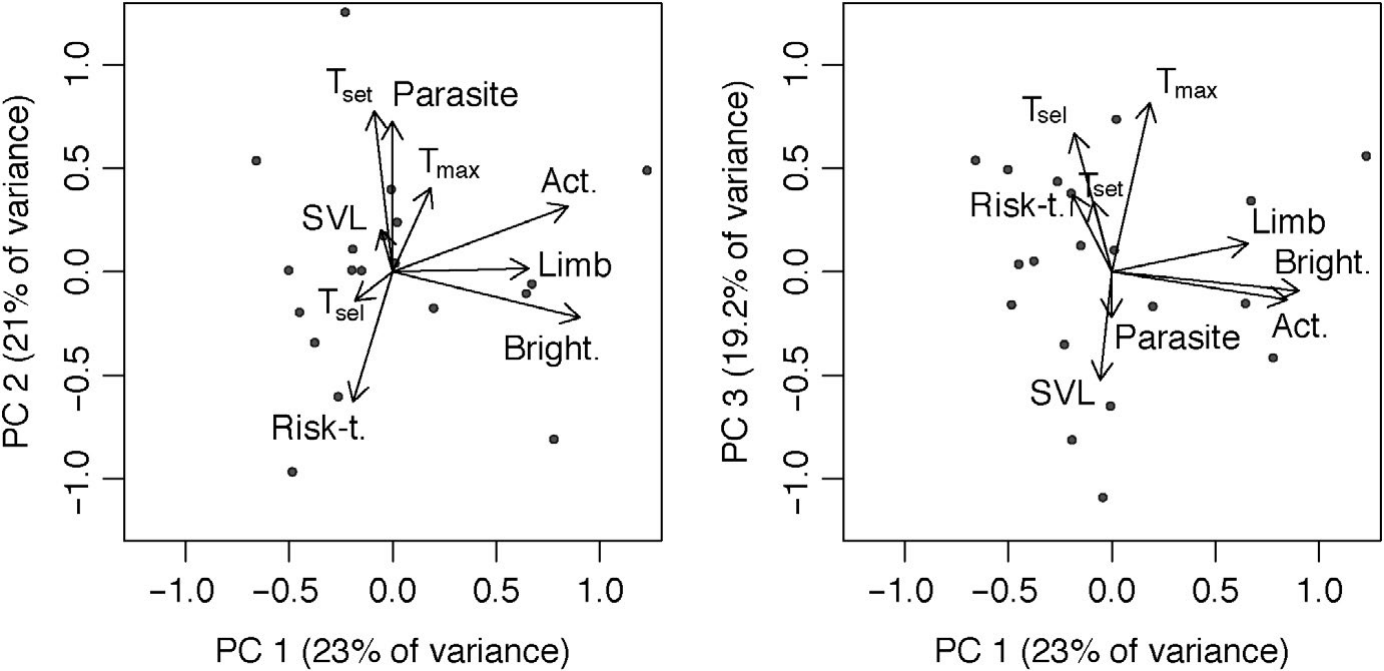
Principal component analysis biplots in rotated space for the 19 individuals (represented as dots). Filled circles represent individual scores and arrows represent factor loadings for original variables in each one of the PCs. Individual scores were divided by 2 for visualizing purposes. Act = activity; Risk‐t = risk‐taking; T_sel_ = median of selected body temperatures; *T*_set_ = set point range; *T*_max_ = thermal voluntary maximum; SVL = snout–vent length, Limb = relative hind limb length; Parasite = blood parasite infection; Bright = dorsal brightness

Our LMM revealed significant differences in selected body temperatures among individuals (χ^2^ = 358.19, *df = *1, *p < *.001; Figure 2), indicating individually variable thermoregulatory strategy. However, results indicated no significant effect of time of day or date (time of day: *F_2,19.01_* = 0.3, *p = *.74; date: *F_1,19_* = 1.89, *p = *.19; time of day × date: *F_1,19_* = 0.06, *p = *.80). Our PCA resulted in three PCs explaining 63.21% of total variation (Table 1). PC1 explained 23% of the total variation and had excellent positive loadings from activity (0.85) and dorsal brightness (0.91) and a good positive loading from relative hind limb length (0.66) (Figure 3). This indicates a gradient from inactive, short‐legged, and dull‐colored males toward active, long‐legged, and brightly colored males. PC2 explained 20.98% of total variation and had an excellent positive loading from T_set_ (0.77) (note that it is a variable describing precision; hence, low values mean high precision), a very good positive loading from parasite load (0.73), and a good negative loading from risk‐taking (−0.63) (note that risk‐taking is a latency variable; hence, low values mean high risk‐taking) (Figure 3). This indicates a gradient from lowly parasitised precise thermoregulators with low risk‐taking toward highly parasitized imprecise thermoregulators with high risk‐taking. PC3 explained 19.23% of total variation and had a good positive loading from T_sel_ (0.67), an excellent positive loading from T_max_ (0.81), and a fair negative loading from SVL (−0.52), indicating a gradient from large males with low preferred body temperature to small males with high preferred body temperature (Figure 3).

**Table 1:** Factor loadings of principal component analysis ran on thermal‐ and individual state‐related traits

	PC1	PC2	PC3
Activity	0.85^a^	0.32	−0.14
Risk‐taking	−0.19	−0.63^c^	0.37
*T*_sel_	−0.18	−0.14	0.67 ^c^
*T*_set_	−0.09	0.77 ^a^	0.34
*T*_max_	0.18	0.4	0.81 ^a^
SVL	−0.06	0.2	−0.52^d^
Relative hind limb length	0.66 ^c^	0.02	0.14
Blood parasite infection	−0.002	0.73^b^	−0.22
Dorsal brightness	0.91 ^a^	−0.22	−0.09
% of variance explained	23	20.98	19.23
Total variance explained	63.21		

Note

Traits with a loading ≥ 0.45 were considered to contribute to a PC. *T*_sel_ = median of selected body temperatures; *T*_set_ = set point range; *T*_max_ = thermal voluntary maximum; SVL = snout–vent length.

^a^ Excellent.

^b^ Very good.

^c^ Good.

^d^ Fair; according to Tabachnick and Fidell (2014).

## 4 DISCUSSION

In the present paper, in line with accumulating empirical research in various poikilotherm taxa (Cerqueira et al., 2016; Goulet, Thompson, & Chapple, 2017; Rey et al., 2015; Stapley, 2006), we show that there is a link between risk‐taking personality and thermoregulatory strategy in adult males of *I. cyreni*. However, the link between risk‐taking personality and behavioral thermoregulatory strategy is only moderate, while totally absent regarding activity, in our model system. It has been suggested that linkage between behavior and various aspects of individual physiology is complex and affected by biotic and abiotic factors (Careau et al., 2015; Killen, Marras, Metcalfe, McKenzie, & Domenici, 2013; Le Galliard, Paquet, Cisel, & Montes‐Poloni, 2013), and thus, we can only speculate about the biological background behind the patterns presented here. Previous results on *I. cyreni* (Horváth, Martín, et al., 2017; Horváth, Rodríguez‐Ruiz, Martín, López, & Herczeg, 2019) and passerines (see Dingemanse, Both, Drent, & Tinbergen, 2004; Garamszegi et al., 2015) indicate that drastic changes in behavioral strategy could occur not just between years, but also among seasons within the same year. Thus, given that our study was conducted on adult males of *I. cyreni* during the seasonally limited breeding period, we cannot exclude the possibility that the moderate association between risk‐taking personality and behavioral thermoregulatory strategy was the outcome of the different reproductive state (e.g., variation in plasma testosterone levels; see Martín & López, 2010) of the studied individuals, or the ecological conditions during this time of year. In any case, additional, long‐term research involving female specimens and measures in other seasons are needed to verify the true nature of this link in *I. cyreni*.

Both activity and risk‐taking personality and behavioral thermoregulatory strategy are associated with various state‐linked traits. Links between coloration and personality have been reported in birds and reptiles several times (Ibáñez, Pellitteri‐Rosa, Sacchi, López, & Martín, 2016; Mafli, Wakamatsu, & Roulin, 2011; Mateos‐González and Senar, 2012; Williams, King, & Mettke‐Hofmann, 2012). In our study, males with brighter dorsal coloration and relatively longer hind limbs were more active. In many lacertid lizards, brightness is considered to be an honest signal because the expression of brighter coloration has physiological costs that only males of higher quality (i.e., higher trait value in potentially fitness‐linked traits; see Wilson & Nussey, 2010) and condition can afford (Bajer, Molnár, Török, & Herczeg, 2010; Kopena et al., 2014; Lisboa, Bajer, Pessoa, Huber, & Costa, 2017; San‐José and Fitze, 2013). Although the exact information content of color signals in *I. cyreni* is not entirely known (Cabido, Galán, López, & Martín, 2009; López, Martín, & Cuadrado, 2004), the species is polygynandric—older, territorial males express turquoise coloration and are preferred by females (Aragón, López, & Martín, 2004; Martín & López, 2013). Also, males with highly saturated green dorsal coloration have higher reproductive success (Salvador, Diaz, Veiga, Bloor, & Brown, 2008). Limb length, in addition, was shown to be strongly correlated with sprint speed (i.e., locomotor performance; Bauwens, Garland, Castilla, & Van Damme, 1995; Vanhooydonck, Van Damme, & Aerts, 2001), which is an important component of life‐history trade‐offs and a suitable proxy for individual quality (Garland, 1984; Husak, Ferguson, & Lovern, 2016; Irschick, Meyers, Husak, & Le Galliard, 2008; Le Galliard, Clobert, & Ferrière, 2004; Winchell, Maayan, Fredette, & Revell, 2018). Hence, males with brighter coloration and longer hind legs could be seen as more attractive, higher‐state individuals. Being more active may allow these males to gain access to territories with abundant resources, while they are most likely protected from predators due to their state (i.e., increased sprint speed). Hence, our results potentially reflect some form of state‐dependent safety, where individuals with higher state are expected to have high behavioral activity for their benefit, because their high state allows them to deal with the increased risk (Luttbeg & Sih, 2010).

Males with high blood parasite infection rate were shown to be imprecise thermoregulators and more risk‐prone than healthier conspecifics. Similarly to previous results (Horváth et al., 2016), this pattern is in line with the asset protection principle (see Clark, 1994), which predicts individuals with low reproductive value to be risk‐prone. *Karyolysus* and *Schellackia* (family Karyolysidae, formerly Haemogregarinidae, suborder Adeleorina, subclass Coccidiasina, phylum Apicomplexa) are intracellular protozoans infecting *I. cyreni* and other reptiles as intermediate hosts (Amo, López, & Martín, 2005; Bouma, Smallridge, Bull, & Komdeur, 2007; Caudell, Whittier, & Conover, 2002; Garrido, Pérez‐Mellado, & Cooper, 2014; Sagonas, Rota, Tsitsilonis, Pafilis, & Valakos, 2016; Smith, Desser, & Martin, 1994; Veiga, Salvador, Merino, & Puerta, 1998). Although infection by Karyolysidae usually does not affect the host's survival directly (Megía‐Palma et al. 2018), blood parasite load in *I. cyreni* has a negative effect on body condition during the mating season (Amo et al., 2004). A similar negative association between infection rate and thermoregulatory precision was reported by Paranjpe et al. (2014) in side‐blotched lizards (*Uta stansburiana*) infected by malaria (*Plasmodium mexicanum*). However, considering the correlative nature of our study, there are several alternative mechanisms explaining the pattern found here. As *I. cyreni* lives in a low thermal quality environment with considerable daily fluctuation in ambient temperature (Aguado & Braña, 2014; Jiménez‐Robles & De la Riva, 2019; Monasterio et al., 2009), high thermoregulatory precision is expected. However, maintaining high precision has considerable energy and time commitments (Bowker, 1984; Paranjpe et al., 2014; Sartorius, do Amaral, Durtsche, Deen, & Lutterschmidt, 2002); thus, it is somewhat plausible that the decrease of thermoregulatory precision is the result of the energetic constraints of the blood parasite infection. Parasitic manipulation of thermoregulatory behavior of the hosts is another possible option (see Lafferty & Shaw, 2013; Poulin, 2013). However, there is no information in the literature that Karyolysidae are capable to actively alter the behavior of its hosts. Intuitively, infection should increase both body temperature and thermoregulatory precision of the host (Karsten, Ferguson, Chen, & Holick, 2009; Scholnick, Manivanh, Savenkova, Bates, & McAlexander, 2010), but lizards might choose to maintain lower thermoregulatory precision in order to reduce the development of parasites and the physiological costs of infection (Paranjpe et al., 2014).

Larger males preferred lower temperatures than smaller conspecifics. As reptile growth is indeterminate, these males are probably older than their smaller conspecifics (Kozlowski, 1996; Shine & Charnov, 1992). Active thermoregulation is often interpreted to be costly for small heliothermic lizards, mainly because they are exposed to predators during basking (Herczeg et al., 2008); thus, optimization of basking and selection of basking sites have utmost importance, especially in high‐mountain habitats, where time for attaining appropriate body temperatures is limited (Jiménez‐Robles & De la Riva, 2019; Martín & Salvador, 1993). For example, previous studies indicate that *I. cyreni* usually choose to bask closer to their shelters (e.g., rock crevices; Carrascal et al., 1992; Martín & Salvador, 1997). As small individuals might be less conspicuous to predators than larger ones (Baxter‐Gilbert & Riley, 2018; Martín & López, 2003), they are able to follow a basking strategy resulting in higher body temperatures than larger conspecifics. Previous results in the European adder, *Vipera berus*, suggest that age groups more vulnerable to predation have lower preferred body temperatures, possibly as a result of predator avoidance (Herczeg, Gonda, et al., 2007). Alternatively, as smaller individuals have lower body mass and higher surface‐to‐volume ratio, they absorb heat faster and can reach high body temperatures within shorter time (Carrascal et al., 1992; Herczeg, Török, & Korsós, 2007; Martín & López, 2003).

Taken together, we found a moderate link between risk‐taking personality and behavioral thermoregulatory strategy in adult male *I. cyreni*, which might be the outcome of reproductive state of the individuals or ecological conditions during the breeding season. Thus, we suggest that future manipulative experiments should also involve female lizards and consider other seasons for a better understanding of the link between these behavioral traits. We found various connections between activity and risk‐taking personality and individual state or between behavioral thermoregulatory strategy and individual state, supporting the state‐dependence of both. We conclude that even though there is a relationship between interindividual variation in risk‐taking personality and behavioral thermoregulatory strategy in this lizard species, behavioral variation is primarily affected by individual state.

## CONFLICT OF INTEREST

The authors declare no conflict of interest.

## AUTHOR CONTRIBUTION

**Gergely Horvath:** Conceptualization (equal); Data curation (equal); Formal analysis (equal); Visualization (equal); Writing‐original draft (equal); Writing‐review & editing (equal). **Octavio Jiménez‐Robles:** Conceptualization (equal); Data curation (equal); Formal analysis (equal); Visualization (equal); Writing‐review & editing (equal). **José Martín:** Conceptualization (equal); Funding acquisition (equal); Supervision (equal); Writing‐review & editing (equal). **Pilar Lopez:** Conceptualization (equal); Funding acquisition (equal); Supervision (equal); Writing‐review & editing (equal). **Ignacio De la Riva:** Conceptualization (equal); Funding acquisition (equal); Supervision (equal); Writing‐review & editing (equal). **Gabor Herczeg:** Conceptualization (equal); Funding acquisition (equal); Supervision (equal); Writing‐review & editing (equal).

## ETHICAL APPROVAL

All applicable international, national, and/or institutional guidelines for the care and use of animals were followed. The experiment was performed under license (permit number: 10/024398.9/13) from the Environmental Agency of Madrid Government (“Consejería de Medio Ambiente de la Comunidad de Madrid,” Spain).

## Supporting information

Table S1‐S2

## Data Availability

The data are available on DRYAD (https://doi.org/10.5061/dryad.qjq2bvqdd).
