# Supplementary material for: Linking behavioral thermoregulation, boldness, and individual state in male Carpetan rock lizards
Source: Ecol Evol. 2020 Aug 17;10(18):10230–41. doi: 10.1002/ece3.6685 (PMC7520217; doi:10.1002/ece3.6685)
Supplement: Supplementary file 1 — Table S1‐S2 [file ECE3-10-10230-s001.docx]

Supplementary Table S1. Factor loadings of Principal Component Analysis ran on three dorsal measurements averaged by 5 nm and then by individual to describe dorsal reflectance.

| wavelength | factor loadings |
| --- | --- |
| nm 300 | 0.979 |
| nm 305 | 0.982 |
| nm 310 | 0.984 |
| nm 315 | 0.987 |
| nm 320 | 0.988 |
| nm 325 | 0.988 |
| nm 330 | 0.989 |
| nm 335 | 0.988 |
| nm 340 | 0.992 |
| nm 345 | 0.993 |
| nm 350 | 0.993 |
| nm 355 | 0.995 |
| nm 360 | 0.997 |
| nm 365 | 0.996 |
| nm 370 | 0.995 |
| nm 375 | 0.993 |
| nm 380 | 0.991 |
| nm 385 | 0.987 |
| nm 390 | 0.987 |
| nm 395 | 0.986 |
| nm 400 | 0.985 |
| nm 405 | 0.986 |
| nm 410 | 0.989 |
| nm 415 | 0.992 |
| nm 420 | 0.992 |
| nm 425 | 0.993 |
| nm 430 | 0.992 |
| nm 435 | 0.989 |
| nm 440 | 0.991 |
| nm 445 | 0.991 |
| nm 450 | 0.992 |
| nm 455 | 0.992 |
| nm 460 | 0.990 |
| nm 465 | 0.984 |
| nm 470 | 0.977 |
| nm 475 | 0.980 |
| nm 480 | 0.983 |
| nm 485 | 0.978 |
| nm 490 | 0.969 |
| nm 495 | 0.949 |
| nm 500 | 0.950 |
| nm 505 | 0.975 |
| nm 510 | 0.989 |
| nm 515 | 0.993 |
| nm 520 | 0.995 |
| nm 525 | 0.994 |
| nm 530 | 0.994 |
| nm 535 | 0.994 |
| nm 540 | 0.995 |
| nm 545 | 0.994 |
| nm 550 | 0.994 |
| nm 555 | 0.993 |
| nm 560 | 0.983 |
| nm 565 | 0.986 |
| nm 570 | 0.992 |
| nm 575 | 0.993 |
| nm 580 | 0.987 |
| nm 585 | 0.980 |
| nm 590 | 0.983 |
| nm 595 | 0.991 |
| nm 600 | 0.991 |
| nm 605 | 0.991 |
| nm 610 | 0.994 |
| nm 615 | 0.993 |
| nm 620 | 0.993 |
| nm 625 | 0.994 |
| nm 630 | 0.994 |
| nm 635 | 0.995 |
| nm 640 | 0.993 |
| nm 645 | 0.992 |
| nm 650 | 0.994 |
| nm 655 | 0.996 |
| nm 660 | 0.996 |
| nm 665 | 0.996 |
| nm 670 | 0.996 |
| nm 675 | 0.997 |
| nm 680 | 0.996 |
| nm 685 | 0.995 |
| nm 690 | 0.992 |
| nm 695 | 0.988 |
| nm 700 | 0.984 |
| Total variance explained | 88.4% |

Supplementary Table S2. Anti-image correlation matrix, presenting the negatives of the partial correlation coefficients. T_sel_= median of selected body temperatures; T_set_ = set point range; T_max_ = thermal voluntary maximum; SVL = snout-vent length, Rel. limb = relative hind limb length; Parasite = blood parasite infection; Dorsal bright. = dorsal brightness.

|  | Risk-taking | T_sel_ | T_set_ | T_max_ | SVL | Rel. limb | Parasite | Dorsal bright. |
| --- | --- | --- | --- | --- | --- | --- | --- | --- |
| Activity | 0.52 | -0.40 | -0.41 | 0.02 | -0.50 | 0.24 | -0.19 | -0.86 |
| Risk-taking | - | -0.52 | -0.31 | 0.14 | -0.24 | 0.40 | 0.23 | -0.57 |
| T_sel_ | - | - | 0.58 | -0.49 | 0.31 | -0.36 | -0.16 | 0.60 |
| T_set_ | - | - | - | -0.63 | 0.25 | -0.10 | -0.29 | 0.53 |
| T_max_ | - | - | - | - | 0.13 | -0.13 | 0.24 | -0.14 |
| SVL | - | - | - | - | - | -0.42 | -0.02 | 0.58 |
| Rel. limb | - | - | - | - | - | - | 0.12 | -0.46 |
| Parasite | - | - | - | - | - | - | - | 0.05 |
